# The Effect of Short-Term and High-Intensity Functional Circuit Training on Plasma Lipidome Profiles of People Living with and Without HIV

**DOI:** 10.3390/metabo16010016

**Published:** 2025-12-24

**Authors:** Marcos Yukio Yoshinaga, Flávio Gomez Faria, Adriano de Britto Chaves-Filho, Sayuri Miyamoto, Tania Cristina Pithon-Curi, Giselle Cristina Bueno, Bruno Ferrari Silva, Sidney Barnabé Peres, Solange Marta Franzoi de Moraes

**Affiliations:** 1 Institute of Physical Activity and Sport Sciences, Universidade Cruzeiro do Sul, São Paulo 01506-000, Brazil; flavio.gomezfaria@gmail.com (F.G.F.); tania.pithon-curi@cruzeirodosul.edu.br (T.C.P.-C.); 2 PinguisLab, São Paulo 04119-010, Brazil; 3 Department of Biochemistry, Institute of Chemistry, Universidade de São Paulo, São Paulo 05508-900, Brazil; adrianobcfilho@gmail.com (A.d.B.C.-F.); miyamoto@iq.usp.br (S.M.); 4 Department of Physiological Sciences, Universidade Estadual de Maringá, Paraná 87020-900, Brazil; gibuenogianini@gmail.com (G.C.B.); ferrarisilvab@gmail.com (B.F.S.); sbperes@uem.br (S.B.P.); smfmoraes@uem.br (S.M.F.d.M.)

**Keywords:** lipidomics, functional circuit training, plasma lipids, antiretroviral therapy, lipid species

## Abstract

Background/Objectives: Both HIV infection and antiretroviral therapy contribute to dyslipidemia and abnormal body fat distribution in people living with HIV (PLWH). Exercise training is an effective intervention to protect against these metabolic changes. However, little is known about the mechanisms underlying the impact of exercise training on lipid metabolism in PLWH. This study aimed to comparatively evaluate the effect of high-intensity functional circuit training on the plasma lipidome of PLWH and HIV-negative subjects (control). Methods: PLWH (n = 13) and control (n = 14) were submitted to 8 weeks of exercise training. Body composition, anthropometric, and biochemical parameters were measured. Plasma was obtained in a fasting state for lipidomic analysis. Results: Anthropometric and biochemical parameters revealed lower levels of leptin, HDL-C, body fat %, and BMI combined with elevated aspartate transaminase (AST) and Homeostasis Model Assessment of β-cell function (HOMA_beta) in PLWH when compared to control subjects that persisted from baseline to post-exercise training. Nonetheless, contrasting levels of adiponectin, fasting insulin, and phosphatidylcholine-containing lipids observed at baseline were equalized after training in PLWH. In control subjects, significant reductions in concentrations of triglycerides alongside phosphatidylinositol and glycosylated ceramides were observed post-exercise training. By contrast, PWLH displayed an increase in diglycerides, acylcarnitines, and free cholesterol levels after exercise training, together with decreased concentrations of free fatty acids, cholesteryl esters, and glycosylated ceramides. Conclusions: In addition to specific lipidome alterations in each group, particularly driven by improved insulin resistance in PLWH, this study showed concomitant modulation of several glycerophospholipids and sphingolipids, suggesting health-promoting effects of short-term exercise training. Collectively, these modulated lipid species represent interesting targets for future lipidomic-based studies evaluating not only the effects of exercise training but also the molecular mechanisms resulting in a healthier plasma lipidome profile.

## 1. Introduction

Contemporary antiretroviral therapy (ART) has improved both survival and quality of life of people living with the human immunodeficiency virus (PLWH) [1,2]. Nonetheless, dyslipidemia and lipodystrophy are highly prevalent in PLWH, significantly contributing to an increased risk of developing cardiovascular diseases (CVD) when compared to the general population [3,4,5]. Lipid profile abnormalities have also been associated with the prevalence of other metabolic disorders, such as non-alcoholic fatty liver disease in PLWH, which may in turn increase the risk of cardiovascular events [6,7,8]. Dyslipidemia in PLWH can occur due to certain types of ART, particularly the first-generation protease inhibitors that may lead to metabolic dysfunctions (e.g., high LDL-C, low HDL-C, and insulin resistance [9,10,11]. Therefore, the management of dyslipidemia in PLWH requires close attention, considering the drug-to-drug interaction between ART medications and lipid-lowering therapies [12,13].

Lifestyle interventions through diet and/or prescription of an exercise regimen could represent an effective and complementary alternative to drug-based therapies in PLWH for prevention and treatment of CVD risk. Promising improvements in immune function, gut microbiota, cardiorespiratory fitness, and blood lipids, among others, are reported in short-term interventions with aerobic exercise, resistance exercise, and their combination [14,15,16,17]. There is limited evidence concerning the effects of exercise on blood lipid panels of PLWH [18,19]. In general, the existing data from various protocols demonstrate increased concentrations of HDL-C with or without decreased levels of cholesterol and triglycerides as the major impacts of exercise training.

Lipids play a central role in atherosclerosis, from the formation and progression of atherosclerotic plaque in the protracted subclinical phase to its subsequent disruption at an advanced stage or after a cardiovascular event [20]. By screening a large number of circulating lipids, plasma lipidome profiling by lipidomic analysis may provide insights into the molecular mechanisms underlying atherosclerosis [21,22]. Lipidomic screening of blood lipids has shown promising results in detecting prominent differences in lipidome composition between PLWH and HIV-negative controls [23,24,25,26], which may be useful for risk stratification in chronic diseases. Interestingly, a recent study applied targeted lipidomics to evaluate the effects of supervised and mixed endurance/resistance exercise on the serum lipidome of PLHW and HIV-negative controls, indicating a very distinct response between these populations [27].

Despite the significant advances in the field of lipidomics during recent years, reliable quantification and identification of the human lipidome are still major challenges for clinical translation [28,29]. In large-scale lipidomics studies, targeted methods have been preferred over untargeted approaches due to high sensitivity and throughput in the quantification of a selected list of molecules. Conversely, by facilitating the discovery of lipid species, untargeted methods can provide a more unbiased picture of the plasma lipidome, which, depending on population, is quite variable in composition [30]. The latter, when applied to population studies, however, is largely dependent on software performance, which is currently unable to identify 40 to 60% of ions detected by mass spectrometry [31].

The present study is aimed at describing the effects of 8 weeks of high-intensity functional circuit training on the plasma lipidome of PLWH (n = 13) and HIV-negative subjects (control group; n = 14). For this purpose, a combined untargeted/targeted lipidomic approach was applied to evaluate plasma lipidome profiles of these groups of subjects at baseline and after 8 weeks of exercise training.

## 2. Materials and Methods

### 2.1. Study Design and Participants

Sedentary adult men and women seronegative (HIV−) or diagnosed with HIV (HIV+) were recruited from the Specialized Care Service for STD/AIDS (Serviço de Assistência Especializada in Maringá, Paraná, Brazil). Participants willing to join the study signed the Free and Informed Consent Form and responded to an anamnesis form. The experimental study procedures were approved by the Institutional Research Ethics Committee under the registration no. 3211855 (Universidade Estadual de Maringá). The experimental trial followed the recommendations outlined by the CONSORT statement for non-pharmacological interventions [32]. The clinical trial was registered in the Brazilian Registry of Clinical Trials (UTN: U1111-1231-1846).

The inclusion criteria for PLWH were positive serology for the HIV virus and ART use for at least six months, lack of motor impairment or comorbidity to perform tests and training, lack of regular physical activity in the last six months, over 18 years of age, and a medical certificate for cardiometabolic capabilities to perform physical exercise. The exclusion criteria for both PLWH and HIV-negative controls were diarrhea, nausea, vomiting, or poor oral food intake; systemic infection in the last 30 days; pregnancy; and at least 85% participation during training sessions.

The sample size was calculated using the G*Power software v3.1.9.7 (Düsseldorf, Germany), considering the power of the test (1 − β = 80%). From this estimation, the ideal size of the study was established at 34 participants. The present two-arm, parallel, non-randomized clinical trial evaluated the effect of 8-week exercise training on plasma hormonal and biochemical responses in PLWH relative to HIV-negative subjects [33]. From a total of 37 subjects submitted to an 8-week exercise training program, the present study examined a subset of 27 participants with a focus on plasma lipidomic analysis. The experimental trial followed the recommendations outlined by the CONSORT statements for non-pharmacological interventions [32], and professionals involved in the selection of the groups were not included in the evaluations and interventions, reducing the risk of bias. This clinical trial was registered in the Brazilian Registry of Clinical Trials (UTN: U1111-1231-1846).

### 2.2. Functional Circuit Training Program

To carry out the training, the following materials were used: mats, steps, TRX, flexion support, and a Swiss ball. The activities were presented in order to develop and stabilize the muscles of the central region of the body (abdominal, lumbar, pelvis, and hip), improving strength and muscle power. In addition, exercises involving motor skills such as balance, coordination, gait, agility, and proprioception were used.

The training program lasted eight weeks, distributed in three weekly sessions with a total duration of 5 min of warm-up and 30 min of exercise execution, composed of ten exercise stations (jumping up, jumping jacks, push-ups, squats, lunges, hip raises, burpees, planks, sit-ups, crate jumps, and bilateral back row). Two rounds were held in a circuit format; the execution time and interval between exercises were controlled in the proportion of 2:1 (effort: pause), being 1 min of stimulus and 30 s of rest, followed by 5 min of stretching and cooling down.

### 2.3. Anthropometric Measurements, Blood Plasma Sampling, and Biochemical Analyses

The multifrequency, tetrapolar electrical bioimpedance technique (Inbody^®^, model R20; InBody Co., Seoul, Republic of Korea) was used to measure body composition. The evaluations were carried out in the morning after fasting for at least 12 h. Thereafter, approximately 8 mL of blood was distributed into tubes containing EDTA or heparin, which were centrifuged at 5000× *g* for a period of 10 min. Aliquots of plasma were stored in 1.5 mL tubes and stored at −80 °C until further analysis.

Plasma samples were used to determine the concentrations of glucose, alanine transaminase (ALT), aspartate transaminase (AST), gamma glutamyl transferase (GGT), total cholesterol and triglycerides, and cholesterol content of very low-density (VLDL), low-density (LDL), and high-density (HDL) lipoproteins that were measured by colorimetric methods using kits from Gold Analyze (Belo Horizonte, MG, Brazil). The concentrations of insulin, leptin, and adiponectin were measured by the ELISA technique followed by analysis on a FlexStation3 plate reader (Molecular Devices, LLC, San Jose) using a commercial kit (Invitrogen^®^, Carlsbad, CA, USA) and according to the manufacturer’s recommendations.

Insulin resistance was assessed via Homeostasis Model Assessment of Insulin Resistance (HOMA_IR) using the formula HOMA_IR = Fasting glucose (mg/dL) × fasting insulin (μU/mL)/405 [34]. Values between 2.0 and 2.5 for HOMA_IR indicate healthy individuals, and values higher than 2.5 indicate insulin resistance. The beta cells’ function was evaluated using HOMA_beta via the formula HOMA_beta = 360 × (fasting insulin [μU/mL]/fasting glucose [mg/dL]) − 63. Reference values of HOMA_beta for healthy individuals are between 100 and 150, with those higher than 150 indicating hyperinsulinemia [34]. Insulin sensitivity was also assessed via the quantitative insulin sensitivity check index (QUICKI) using the formula QUICKI = 1/(log fasting insulin [μU/mL] + log fasting glucose [mg/dL]). Results from QUICKI of 0.33–0.36 are observed in healthy individuals, while values below 0.33 indicate insulin resistance [35]. Finally, the insulin resistance in adipocytes was calculated via the Adipo_IR index by the formula Adipo_IR = free fatty acid (mmol/L) × fasting insulin (μU/mL). Adipo_IR values below 10 suggest healthy individuals, whereas those higher than 10 indicate a high likelihood of metabolic syndrome [36].

### 2.4. Lipidomic Analysis

Plasma samples (50 µL) were extracted according to [37] with minor modifications and analyzed by a lipidomic analysis protocol developed by our group [38]. A mix of internal standards (100 µL; Supplementary File, Internal Standards) was spiked into the samples, and afterwards, 200 µL of cold methanol was added, and this mixture was vortexed for 10 s. Subsequently, 1 mL of methyl tert-butyl ether (MTBE) was added, and the mixture was vortexed for 30 s and stirred at 1000 rpm for 1 h at 20 °C. In the following step, 300 µL of water was added to the tubes, which were kept in an ice bath for 10 min, and samples were vortexed (30 s) and centrifuged (10,000× *g* for 10 min at 4 °C). After centrifugation, the supernatant containing the total lipid extract (TLE) was collected and dried in a microtube under N2 gas. The TLEs were resuspended in 100 µL of isopropanol and centrifuged (1500× *g* for 3 min at 4 °C). Aliquots of 5 µL from all samples were collected in a vial and used as a quality control sample, which was injected into the mass spectrometer at the beginning and after 9 experimental samples.

The TLE was injected, and lipid separation was obtained by ultra-high-pressure liquid chromatography (Nexera UHPLC, Shimadzu, Kyoto, Japan) equipped with a CORTECS^®^ (UPLC^®^ C18 column, 1.6 µm, 2.1 mm i.d. × 100 mm). Operating at a flow rate of 0.2 mL/min at 35 °C, the mobile phases consisted of solvent A (water and acetonitrile [60:40]) and solvent B (isopropanol, acetonitrile, and water [88:10:2]). The gradient elution process was initiated at 40 to 100% of solvent B over the first 10 min. The samples were kept at 100% of solvent B from 10 to 12 min and decreased to 40% of solvent B from 12 to 13 min, which was held for 20 min. The UHPLC was coupled to a triple quadrupole time-of-flight mass spectrometry (Triple TOF^®^ 6600, Sciex, Concord, CA, USA), with the electrospray ionization operating at −4.5 kV and 5.5 kV for negative and positive ionization modes, respectively, and cone voltage set at ±80 V. The curtain gas was set at 25 psi, nebulizer and heater gases to 45 psi, and interface heat at 450 °C. The scan range was set to 200–2000 Da.

The Information Dependent Acquisition (IDA) data were analyzed in PeakView software v2.2 (Sciex, Concord, CA, USA). The identity of lipids was based on *m*/*z*, retention time, specific fragments, and/or neutral losses in MS/MS [39,40]. The identification of lipids in the untargeted approach aimed at 70 to 85% coverage of the most abundant ions in IDA. The annotated lipid identities and retention times were then used in the targeted approach, which consisted of peak areas of the precursor ions (±5 mDa) calculated using the MultiQuant software v3.0.3 (Sciex, Concord, CA, USA). The peak areas of compounds of interest were then normalized by the peak area of the corresponding internal standard. The final concentration was given as nmol lipid per µL of plasma (Supplementary File, Lipid Data). The nomenclature of the fatty acyl chains of lipid subclasses is given as X:Y (with X representing the number of carbons and Y the number of double bonds). For sphingolipids, the sphingoid base is expressed as dX:Y and the n-acyl chains as X:Y. It is important to mention that the precise position of the fatty acyl chains in the glycerol backbone of glycerolipids and glycerophospholipids or the sugar moieties of hexosyl ceramides could not be exactly determined using our methods.

### 2.5. Statistical Analysis

The lipidomic analysis results were uploaded into MetaboAnalyst (https://www.metaboanalyst.ca/, accessed on 10 August 2025) [41]. Prior to statistics, the coefficient of variation (CV) was calculated from quality control samples measured throughout the analytical experiments for each lipid reported in this study. Lipid species displaying a CV higher than 20% were excluded from statistics. To achieve a normal distribution, the data were log10-transformed prior to multivariate and univariate analyses. Multivariate analyses were performed by principal component analysis (PCA) and partial least squares discriminant analysis (PLSDA). Both unpaired (to test baseline and post-exercise differences between PLWH and controls) and paired (to test the effects of exercise training: post versus baseline within PLWH and controls) univariate analyses were conducted by *t*-test (*p* < 0.05 for significance and false discovery rate applied). Univariate analyses of the anthropometric and biochemical data were performed using the same approach.

### 2.6. Availability of Data and Materials

The dataset supporting the conclusions of this article is included within the article and its Supplementary Files.

## 3. Results

### 3.1. Clinical Measurements

Lipidomic analysis was performed in a small subset of samples from a non-randomized trial, from which anthropometric and biochemical parameters have been reported elsewhere [42,43,44]. To compare the lipidomic data with these clinical measurements, the major differences between PLWH and control subjects used in this study are described for baseline and post-exercise training. At baseline conditions, PLWH presented significantly lower body fat %, BMI, HDL-C, and leptin, together with higher AST and HOMA_beta, as compared to control subjects, and these parameters were significantly different after exercise training (Table 1). In contrast, higher levels of adiponectin, insulin, HOMA_IR, and Adipo_IR observed in PLWH relative to control subjects at baseline were no longer significant post-exercise (Table 1). Nonetheless, in addition to the differences in several parameters that were maintained from baseline to post-exercise training, levels of total cholesterol were reduced in PLWH as compared to control subjects after exercise training (Table 1).

### 3.2. Plasma Lipidome Diversity

**Figure 1:**
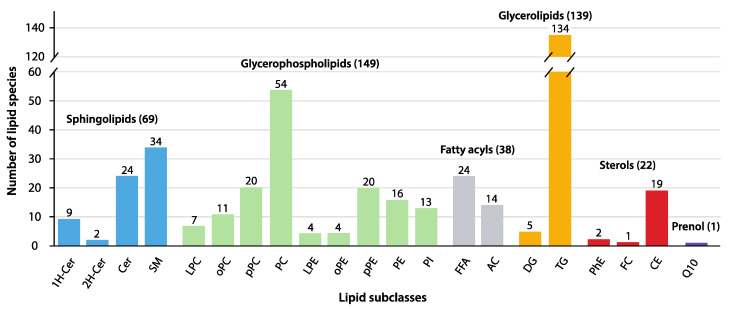
Plasma lipidome diversity displaying the total number of molecular species within each lipid class and subclass (color-coded bars). Please see the main text for the description of lipid subclass abbreviations.

This study identified and quantified a total of 418 individual plasma lipid species distributed into six major lipid classes: glycerolipids, glycerophospholipids, sphingolipids, fatty acyls, sterols, and prenols (Figure 1). As commonly reported in untargeted lipidomic studies, triglycerides (TG) were the most diverse subclass, with 134 lipid species (Supplementary File, Lipid Data). The abundant TG species were followed by phosphatidylcholine (PC; 54 sp), sphingomyelin (SM; 34 sp), unsubstituted ceramides and free fatty acids (Cer and FFA, respectively; 24 sp each), and plasmenyl-PC and -phosphatidylethanolamine (pPC and pPE, respectively; 20 sp each) (Figure 1). Other subclasses composed of more than 10 lipid species included cholesteryl esters (CE; 19 sp), PE (16 sp), acylcarnitines (AC; 14 sp), phosphatidylinositol (PI; 13 sp), and plasmanyl-PC (oPC; 11 sp). Lastly, lipid subclasses with relatively fewer molecular species comprised mono- and di-hexosyl ceramides (1H- and 2H-Cer with 9 and 2 sp, respectively), plasmanyl-PE (oPE; 4 sp), lyso-PC (LPC; 7 sp), lyso-PE (LPE; 4 sp), diacylglycerides (DG; 5 sp), and phytosterol esters (PhE; 2 sp), as well as single species of free cholesterol (FC) and coenzyme Q-10 (Q10).

### 3.3. Comparative Response to Exercise Training in the Lipidome

**Figure 2:**
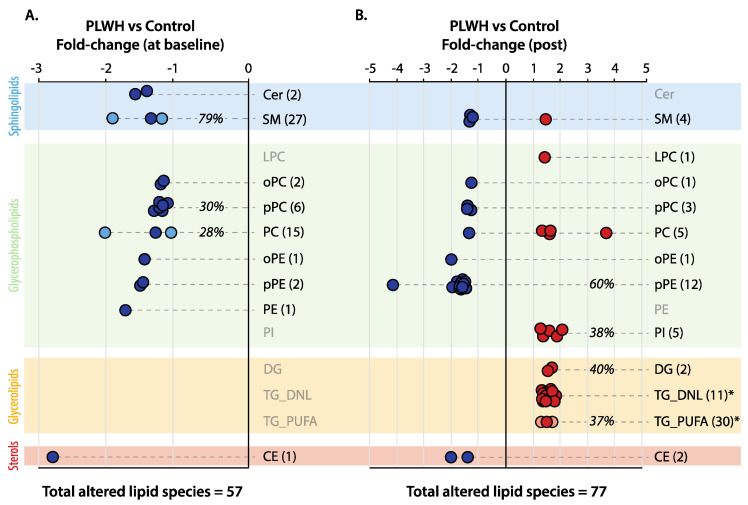
Fold-change values of significantly altered plasma lipid species differentiating PLWH and control subjects at baseline (**A**) and post-exercise training (**B**). Symbols: circles indicate fold-change values of lipid species displaying lower (blue) or higher (red) concentrations in PLWH relative to control subjects. When 15 or more significantly altered lipid species exist within a subclass, a blue or red circle represents an average value delimited by the highest and lowest fold-change values in navy or light red, respectively (e.g., PC in (**A**)). Note that the total number of altered lipid species is given in parentheses for each subclass. Background colors indicate distinct classes of lipids and are described accordingly (left corner). An unpaired *t*-test with *p* < 0.05 for significance was applied for these comparisons. * Note that TG were divided into: (1) those with at least one polyunsaturated fatty acid (PUFA) that must be acquired from the diet and (2) those exclusively composed of saturated and/or monounsaturated fatty acids also sourced by de novo lipogenesis (DNL). Please see the main text for abbreviations.

To examine the differences in plasma lipidome compositions of PLWH and control subjects, an unpaired *t*-test coupled to fold-change analysis was performed at baseline and after training (Figure 2). Of note, with only a few exceptions, most plasma lipids exhibited a <2-fold change. At baseline, 57 plasma lipid species were found in reduced concentrations in PLWH relative to control subjects. These altered lipids were mainly represented by pPC (6 sp), PC (15 sp), and SM (27 sp), comprising, respectively, 30, 28, and 79% of all species within these subclasses monitored in this study (Figure 2A). In comparison, after 8 weeks of high-intensity training, a relatively higher number of 77 plasma lipid species were found in altered concentrations between groups (Figure 2B). A major reduction was observed in 12 pPE species (representing 60% of all pPE species described in this study), with one of them exhibiting a >4-fold change between PLWH and control subjects. Interestingly, most of the down-regulated lipids in PLWH as compared to control subjects at baseline (particularly pPC, PC, and SM) were normalized after exercise training, with only a few exceptions (Figure S1). Plasma lipid species displaying increased concentrations in PLWH relative to control subjects after exercise training represented 70% of all altered species (Figure 2B) and were represented by TG (41 sp), PI (5 sp), and DG (2 sp). Of note, one out of four PC species displayed >3-fold higher concentration in PLWH as compared to control subjects post-exercise. Clearly, however, these results reflected the effects of exercise training in the plasma lipidome of control subjects (e.g., decreased concentrations of TG, PI, and PC) and PLWH (e.g., decreased concentrations of CE and increased levels of DG) relative to baseline, which will be described next.

### 3.4. Multivariate Analysis of Lipidomic Differences

To examine the effects of exercise training in PLWH and control subjects, both multivariate and univariate analyses were performed with paired data. Unsupervised and supervised multivariate analyses were performed by principal component analysis (PCA) and partial least squares discriminant analysis (PLSDA), respectively. Both PCA and PLSDA revealed a moderate segregation of groups in score plots (Figure S2A,C). With a total of 64.3% and 59.2% explanation of data variability based on the two principal components of the PC and PLSDA, respectively, a consistent spatial segregation of exercise training versus baseline conditions was achieved in score plots (Figure S2A,C). The loading plots showed that concentrations of glycerolipids, particularly TG, were dominant features regulating negative and positive values of component 1, respectively, for PCA and PLSDA (Figure S2B,D). Positive values in component 2 of the PCA and PLSDA were determined by a handful of glycerophospholipids and 2H-Cer (d18:1/24:1), whereas negative values were driven by glycerolipid concentrations (Figure S2B,D). It is worth mentioning that exercise training promoted a clear segregation of groups in PCA and PLSDA, particularly for control subjects (Figure S2A,C). Of note, suggesting a more pronounced reduction in plasma TG concentrations, most of the samples from the control group post-exercise training scored positive and negative for component 1 of PCA and PLSDA, respectively, contrasting with samples from the PLWH group.

### 3.5. Alterations in Lipid Species from Control Subjects

**Figure 3:**
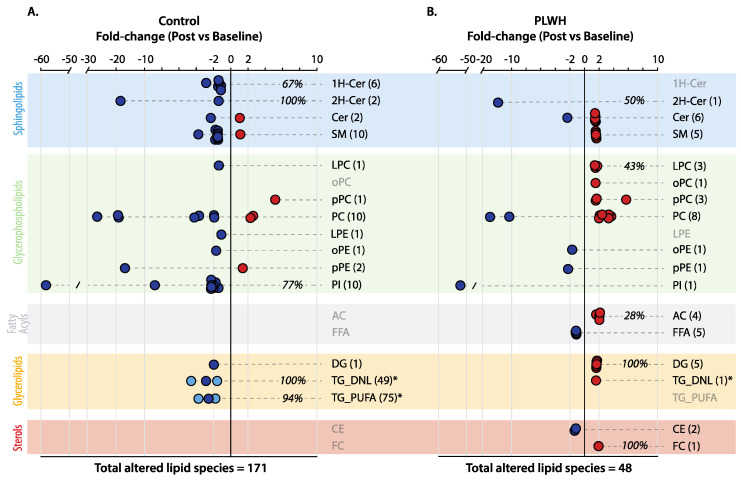
Fold-change values of significantly altered plasma lipid species differentiating post-exercise and baseline in control subjects (**A**) and PLWH (**B**). Symbols: circles indicate fold-change values of lipid species displaying lower (blue) or higher (red) concentrations in post-exercise relative to baseline. When more than 10 significantly altered lipid species exist within a subclass, a blue circle represents the average value delimited by the highest and lowest fold-change values in navy (e.g., TG_PUFA in (**A**)). Note that the total number of altered lipid species is given in parentheses for each subclass. Background colors indicate distinct classes of lipids and are described accordingly (left corner). A paired *t*-test with *p* < 0.05 for significance was applied for these comparisons. * Note that TG were divided into: (1) those with at least one polyunsaturated fatty acid (PUFA) that must be acquired from the diet and (2) those exclusively composed of saturated and/or monounsaturated fatty acids also sourced by de novo lipogenesis (DNL). Please see the main text for the description of lipid subclasses’ abbreviations.

Exercise training resulted in distinct lipidome profile alterations in PLWH and control subjects according to paired univariate analysis (Figure 3). In control subjects, exercise training led to significantly altered concentrations of 171 lipid species, most of them displaying reduced concentrations (164 sp) and 80% of them with >2-fold change as compared to baseline (Figure 3A). The data highlight that several TGs and PIs (representing 92 and 77% of all monitored species, respectively), along with 1H- and 2H-Cer (67 and 100% of total species, respectively), displayed reduced concentrations relative to baseline. Other modulated lipid subclasses with a relatively high number of species included PC (9 sp) and SM (10 sp), with most of them displaying reduced concentrations in post-exercise as compared to baseline. Of note, almost all PC species displayed > 2-fold change, with three of them reaching a 20-fold reduction post-exercise (Figure 3A). In addition to PC, relatively high negative fold-change values were observed for two PIs (9- and 59-fold) and a single species of pPE (17-fold) and 2H-Cer (19-fold).

### 3.6. Comparative Lipidomic Changes After Exercise in PLWH

As compared to control subjects, the modulation of plasma lipidome profiles by exercise training in PLWH was much less extensive, 47 species in total, with only 20% of them displaying >2-fold change (Figure 3B). Unlike the alterations observed in control subjects, increased concentrations of plasma lipids prevailed after exercise training (33 species in total), including FC, DG, LPC, and AC, representing, respectively, 100, 100, 43, and 28% of all species monitored in this study. In addition, several plasma species of PC and SM in PLWH that were found in lower concentrations relative to control subjects at baseline (Figure 2A,B) displayed increased levels after exercise training (Figure 3B). Despite exhibiting a low number of species with significant reductions, the concentrations of five FFAs (averaging 1.2-fold) and two CE (with 1.2- and 1.3-fold) were decreased after exercise training. In summary, a Venn diagram revealed very few overlaps in the modulation of the plasma lipidome by exercise training in PLWH and control subjects, suggesting a distinct scenario with most of the changes linked to increased and decreased concentrations of unique lipid species, respectively (Figure S3). Interestingly, however, an overlap of four species with >10-fold reduction appeared as strong indicators of exercise training relative to baseline for both PLWH and control subjects, respectively: 11- and 27-fold for PC (16:0/16:1), 17- and 19-fold for PC (18:1/22:6), 51- and 59-fold for PI (16:0/18:1), and 14- and 19-fold for 2H-Cer (d18:1/24:1) (Figure S4).

### 3.7. Biochemical and Anthropometric Measurements Associated with Lipid Species

**Figure 4:**
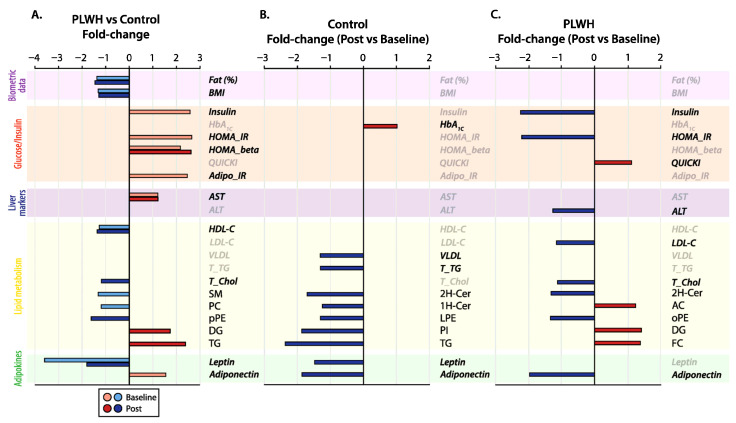
Fold-change variation in significantly altered clinical measurements and lipid subclasses. (**A**) Unpaired *t*-test (*p* < 0.05) of PLWH versus control subjects with horizontal bars representing positive and negative fold-change values at baseline (light red and navy, respectively) and post-exercise (red and blue, respectively); (**B**,**C**) Paired analysis of individual groups in response to high-intensity training with horizontal bars representing positive (red) and negative (blue) fold-change values in post-exercise relative to baseline. Background colors indicate the categories of parameters evaluated (left corner). Please see the main text for the description of lipid subclasses and clinical measurement abbreviations.

Furthermore, we sought to compare the effects of exercise training on the plasma lipidome to anthropometric and biochemical measurements, and for this purpose, the summed concentrations of species within each subclass were calculated (Figure 4). This analysis revealed that PLWH exhibited lower concentrations of PC and SM relative to control subjects at baseline (as previously mentioned for several lipid species). Plasma lipidome differences were found in concert with higher levels of adiponectin, insulin, HOMA_IR, and Adipo_IR, with the latter three exhibiting > 2-fold difference in PLWH as compared to control subjects at baseline (Figure 4A). Interestingly, the latter differences were equalized post-exercise training. Eight weeks of exercise training had no major effects on body fat %, BMI, HDL_C, and AST in PLWH as compared to control subjects (Figure 4A; Table 1). However, exercise training attenuated the reduction in leptin from 3.6-fold at baseline to 1.8-fold in PLWH relative to control, while a slight increase in HOMA_beta (2.2- to 2.6-fold) was noticed (Figure 4A). Regarding plasma lipidome profiles, exercise training led to increased concentrations of DG and TG in PLWH as compared to control subjects, as well as reduced pPE and total cholesterol levels estimated with traditional clinical measurements.

Clinical and lipidome profile alterations in response to exercise training by paired analysis revealed a common trend among PLWH and control subjects: decreased concentrations of 2H-Cer and adiponectin, with similar fold-change values, as compared to baseline conditions (Figure 4B,C). Despite these common alterations, most of the features significantly modulated by high-intensity exercise training were peculiar to each group. In control subjects, decreased concentrations around 2-fold were observed for TG and PI that corresponded to modest reductions of <1.5-fold in LPE and 1H-Cer together with leptin, VLDL, and total TG post-exercise relative to baseline (Figure 4B). Moreover, a small increment in HbA1c was also noticed in control subjects after training. In PLWH, major alterations in post-exercise relative to baseline conditions were linked to biochemical parameters, particularly the >2-fold reduction in insulin and HOMA_IR (Figure 4C). In addition, modest decreases in ALT, LDL-C, total cholesterol, and oPE levels were accompanied by increased DG, FC, and AC concentrations and QUICKI in PLWH post-exercise.

## 4. Discussion

This study aimed to investigate the impact of high-intensity functional circuit training for 8 weeks, corresponding to 24 sessions, on the plasma lipidome composition of PLWH as compared to control subjects. In addition to a relatively low number of participants, this non-randomized trial also involved a heterogeneous population regarding clinical conditions presented at baseline, both inter- and intra-groups (Table 1). The most significant distinction between PLWH and control subjects at baseline was the contrasting levels of adiponectin, fasting insulin, and values of HOMA_IR and Adipo_IR, which were all equalized after 8 weeks of exercise training. Nonetheless, lower levels of leptin, HDL-C, body fat %, and BMI, combined with elevated AST and HOMA_beta in PLWH relative to control subjects, continued from baseline to post-exercise. Thus, our study involved a control group comprising a high proportion of overweight individuals and a PLWH group presented with severe insulin resistance at baseline. Considering this limitation, we will first focus on the major distinctions in plasma lipidome composition between PLWH and control subjects.

One of the most evident features differentiating the plasma lipidome profiles of PLWH and control subjects at baseline was the relatively lower plasma concentrations of several species of PC, pPC, and SM (Figure 2A), which are all particularly dependent on choline metabolism. Such a negative association of plasma choline-based phospholipids with HIV infection has been reported in earlier lipidomic investigations [23,24,25,26]. Choline is deemed an essential nutrient, and the liver is a major site of choline metabolism, as it can be irreversibly oxidized to betaine and plays a central role in the synthesis of hepatic phospholipids [45]. The predominant CDP-choline pathway for de novo hepatic synthesis (accounting for 70% of liver PC) is largely dependent on choline availability, and PC is the precursor of other choline-containing phospholipids such as the acyl-monoethers oPC and pPC together with LPC and SM [46]. In retrospect, liver PC is required for the packaging and export of very-low-density lipoprotein (VLDL) [47,48,49], as demonstrated in both rodent models and human studies showing that choline depletion leads to the development of fatty liver through hepatic TG accumulation [50]. Liver diseases are the number one cause of non-AIDS morbidity and mortality in PLWH [51,52], and their prevalence in the HIV-positive population is considerably higher than in the general population [53,54]. Levels of AST were higher in PLWH than control subjects both at baseline and post-exercise training, and although not exclusively produced in the liver, AST is measured routinely to evaluate clinical conditions of the liver [55]. While the main causes of liver diseases in PLWH are still debatable, including the side effects of combined ART [7], there exists recent evidence for significant alterations in the gut–liver axis [56]. For instance, resulting from increased gut microbial choline metabolism, plasma metabolites such as trimethylamine-N-oxide are elevated and associated with atherosclerosis in PLWH [57,58]. Elevated gut microbial choline metabolism leading to choline depletion in PLWH may also trigger hepatic steatosis [56], as reported for fatty liver diseases not related to HIV [50,59]. Thus, lower levels of plasma choline-containing phospholipids in PLWH relative to control subjects at baseline may indicate altered gut microbial choline metabolism and/or fatty liver, in both ways suggesting hepatic TG accumulation. Our findings revealed that exercise training in PLWH improved fasting FFA and particularly insulin levels that most likely contributed to the significant decrease in ALT levels, a more specific marker of the liver’s health status than AST [55,60]. Correspondingly, differences in plasma concentrations of choline-containing phospholipids in PLWH relative to control subjects were considerably attenuated after 8 weeks of high-intensity functional circuit training.

Another intriguing finding was the reduction in several species as well as total concentrations of acyl-monoether PE, particularly pPE or plasmalogen, exclusively after exercise training in PLWH relative to control subjects (Figure 4A). The role of circulating plasmalogens in health and disease, particularly in aging, is relatively well studied [61]. Thus far, however, the response of circulating plasmalogens to exercise training in humans has not been thoroughly investigated. While upon synthesis, plasmalogens are promptly secreted by the liver [62], their reduced plasma concentrations post-exercise training may indicate decreased lipoprotein secretion in PLWH relative to control subjects. Alternatively, a significant reduction in LDL-C and total cholesterol was observed in PLWH, but not in control subjects, after exercise training; therefore, decreased concentrations of pPE could also be linked to a reduction in LDL particles.

Our study revealed distinct plasma lipidome alterations between PLWH and control subjects in response to high-intensity functional circuit training regardless of weight loss (Figure 3). In control subjects, the molecular signatures of exercise training were linked to significant reductions in plasma concentrations of almost all TG alongside PI, 1H- and 2H-Cer. In contrast, PLWH displayed a significant decrease in plasma concentrations of FFA, CE, and 2H-Cer together with increased concentrations of DG, AC, and FC after exercise training relative to baseline. To a certain extent, these results were consistent with traditional lipid panels, revealing reduced concentrations in total TG and VLDL for control subjects and decreased concentrations of LDL-C and total cholesterol for PLWH after exercise training.

Of note, the only biochemical parameter concurrently modulated in both groups after exercise training was the reduced levels of adiponectin. In general, chronic exercise training, particularly involving significant weight loss, leads to opposing responses of the two major adipokines: increased adiponectin combined with decreased leptin [63,64]. In the absence of weight loss after exercise, reduced adiponectin levels might reflect improved hepatic clearance rather than secretion by the adipose tissue [65,66]. This is the case of lean individuals with non-alcoholic fatty liver disease that display high adiponectin levels relative to controls [67], similar to PLWH versus control subjects at baseline, indicating delayed clearance instead of increased adiponectin secretion, such as in obesity.

Despite the low number of mutual plasma lipidome alterations after exercise training, an interesting finding was related to the decreased concentrations of seven lipid species, with four of them displaying >10-fold change in both groups. Thus, overlapping reductions in 2H-Cer (d18:1/24:1), PC (16:0/16:1), PC (18:1/22:6), and PI (16:0/18:1) with substantially high fold-change values stand out as molecular signatures of potentially beneficial effects of exercise training in the plasma lipidome of PLWH and control subjects. For instance, lactosyl ceramides, such as the 2H-Cer (d18:1/24:1), have been considered markers of inflammation and atherosclerosis [68], and in plasma samples their elevated concentrations were associated with both diabetes and CVD risks [69,70].

A concomitant modulation of plasma TG and PI, as observed only in control subjects after exercise training, has also been recently reported by two independent investigations from our group. The latter studies provided evidence for increased plasma TG-PI concentrations during postprandial lipemia elicited by a high-fat meal in healthy women [38] and after 6 weeks of statin washout in subjects with familial hypercholesterolemia [71]. Importantly, reduced postprandial lipemia and fasting TG concentrations are consistently reported as hallmarks of both exercise and statin use [72,73,74]. Given the transient episode of hypertriglyceridemia that occurs several times each day [75,76], lowering postprandial lipemia by chronic exercise training is likely to be even more relevant than fasting TG concentrations in the context of cardiovascular diseases. In contrast to a lack of changes in body weight, insulin, or HOMA_IR from baseline to post-exercise, reduced levels of adiponectin and leptin were noticed after 8 weeks of high-intensity exercise training in control subjects. Whether the reduction in two of the most important adipokines is directly or indirectly associated with the plasma TG-lowering effects of exercise training is currently unknown. Although supported by our previous data, a direct link between plasma TG and PI concentrations to VLDL and/or postprandial TG-rich lipoprotein levels in response to exercise still needs further mechanistic assessments.

While decreased fasting TG concentrations appear as one of the most often reported effects of chronic exercise training on circulating lipids [77,78,79], our data showed no apparent evidence for significant TG pool alterations in PLWH despite improved fasting insulin levels, HOMA_IR, and QUICKI. A reasonable explanation for the lack of TG pool alterations in PLWH could be related to tissue-specific improvement in insulin resistance. On the one hand, reduced fasting insulin by endurance exercise training in healthy men was demonstrated to significantly increase lipoprotein lipase activity [80], which would increase VLDL catabolic rates, thus lowering plasma TG concentrations. On the other hand, a marked decrease in fasting insulin levels could also result in increased hepatic VLDL secretion [81] and increased production rates as observed after exercise training [82]. Thus, a delicate balance between the VLDL’s production, secretion, and catabolism, likely influenced by hepatic and/or peripheral insulin resistance/sensibility status, could explain the distinct plasma TG concentration responses to exercise training in PLWH and control subjects.

By far the most remarkable effect of exercise in PLWH was the slight but significant reductions in both total cholesterol and LDL-C, features that were not achieved by control subjects and are seldom apparent in short-term interventions [77,83]. The exact mechanism for such a reduction in LDL-C and total cholesterol levels is not firmly established, but there is evidence for the role of LDL receptor—via reduced levels of proprotein convertase subtilisin/kexin type 9 (PCSK9) prompted by exercise training—for the hepatic clearance of cholesterol-rich particles [84]. Consistent with these data, two cholesteryl esters (CE) were found in reduced concentrations, while a slight increase in free cholesterol (FC) was also observed. The latter effects could be attributed to the unusual nature of nonalcoholic fatty liver diseases, where the hepatic synthesis of FC may be uncoupled to CE production [85]. It is worth mentioning that reductions in LDL-C and total cholesterol occurred in concert with lower levels of ALT in PLWH after exercise training.

Although to a lesser extent as compared to LDL-C and total cholesterol, plasma lipidome responses to 8 weeks of exercise training in PLWH could also be perceived as health-promoting alterations. For instance, concentrations of several FFA species were found to be modestly reduced in PLWH post-exercise training. Fasting FFA concentrations are not only elevated in obesity, insulin resistance, non-alcoholic liver diseases, and type 2 diabetes mellitus, but also deemed a critical risk factor for cardiovascular disease development [86]. Such reductions in fasting FFA concentrations with chronic exercise training have been reported by several studies [87,88,89], although it is currently unclear whether a broad range of exercises (e.g., endurance or resistance training) would invariably lead to similar outcomes [86]. In contrast to FFA, the concentrations of several short- to medium-chain acylcarnitines (AC; 2:0, 8:0, 10:0, and 14:0) increased in PLWH after exercise training. Generally, increased concentrations of AC are associated with insulin resistance and diabetes, reflecting incomplete mitochondrial beta-oxidation and efflux of these compounds into the circulation [90]. However, increased plasma concentrations of AC are also observed in metabolic challenges linked to high lipolytic rates and thus elevated substrate availability for beta-oxidation, such as in exercise and starvation [91,92]. Earlier studies have provided strong evidence for the release of acetylcarnitine (i.e., AC (2:0)) [93,94] during high-intensity exercise training, whereas modern metabolomic investigations have linked production and efflux of medium-chain AC to beneficial functions of muscle tissue during moderate-intensity exercise training [95].

Lastly, PLWH after exercise training displayed higher plasma concentrations for all DG species reported in this study. Increased concentrations of DG and ceramides in both skeletal muscle and liver are deemed putative mediators of lipid-induced insulin resistance [96,97]. On the other hand, increased DG plasma concentrations are less obvious but more likely reflect vascular metabolism (e.g., higher lipolytic activity) [98] rather than tissue modifications. Such an increase in plasma DG levels together with decreased TG concentrations were the main findings of a recent targeted lipidomic study evaluating the effects of endurance/resistance exercise in PLWH for 24 weeks [27]. Interestingly, the same effect in PLWH was not apparent in their control subjects, which was attributed to differences in lipid metabolism and fatty acid oxidation between populations.

This study has several limitations, including the varying clinical conditions that could not be fully matched with control subjects. From a clinical perspective, PLWH displayed significantly lower BMI, body fat %, leptin, and HDL-C levels than control subjects at baseline, while presenting higher levels of adiponectin, AST, fasting insulin, and HOMA and Adipo_IR indexes. Although the latter parameters suggest to some degree hepatic inflammation and severe insulin resistance for PLWH, with improved outcomes after exercise training, insulin sensitivity status was not precisely evaluated (e.g., by hyperinsulinemic-euglycemic clamp). Moreover, even though a few non-specific markers, such as AST and ALT, were employed in this non-randomized trial, hepatic steatosis was not assessed by accurate imaging methods (e.g., ultrasound or magnetic resonance) [99], which ultimately restrained our discussion on liver status to general markers. Despite the high variability in clinical parameters, including inter- and intra-group discrepancies, and possible confounding factors, such as changes in diet composition and medication, the lipidomic data revealed a surprisingly high number of lipid species modulated by exercise training. More importantly, distinct plasma lipidome responses resulted in very specific molecular signatures for each group. While the improvement in VLDL and TG clearance observed in control subjects might be considered a classic effect of exercise training in overweight individuals, the responses of PLWH have likely revolved around the improvement in insulin sensitivity and hepatic steatosis. It is uncertain, however, whether an extended program of exercise training for PLWH (i.e., more than 8 weeks) would in fact produce a reduction in VLDL and TG comparable to that detected in control subjects, as well as to a 24-week program as reported by Bowman [27].

In addition to the specific lipidome alterations of each group, this study revealed concomitant modulation in several lipid molecular species, suggesting health-promoting effects of short-term high-intensity functional circuit training. Some of those overlapping plasma lipid species included increased concentrations of PC (17:0/20:4), PC (18:1/20:4), PC (20:3/20:4), and pPC (p17/20:4), all of them linked to a chain of arachidonic acid, together with SM (d18:2/24:1). In contrast, commonly modulated plasma lipid species displaying reduced concentrations included the previously mentioned 2H-Cer (d18:1/24:1), PC (16:0/16:1), PC (18:1/22:6), and PI (16:0/18:1), displaying high fold-change values as well as pPE (p18/18:1), oPE (o18/20:4), and Cer (d18:1/18:0). Collectively, these commonly modulated lipid species represent interesting targets for future lipidomic-based studies evaluating not only the effects of exercise training but perhaps also the molecular mechanisms ensuing a healthier plasma lipidome profile. Similarly, a selected number of plasma lipid species was recently reported as potential markers of favorable and deleterious cardiometabolic conditions in the context of aging in both women and men [100].

## 5. Conclusions

In conclusion, lipidomic analysis examining lipids at the molecular species level may strengthen our ability to understand plasma lipidome variations in health and disease, perhaps leading to an improved patient stratification in clinical studies.

## Figures and Tables

**Table 1:** Anthropometric and biochemical parameters expressed as average ± standard deviation at baseline and post-exercise training in PLWH and control subjects.

	Control (n = 13)		PLWH (n = 14)	
**Age (years)**	37.76 ± 14.1		40.7 ± 16.0	
**Female (%)**	77		57.2	
**Anthropometric measurement**				
	**Baseline**	**Post**	**Baseline**	**Post**
**Weight (kg)**	82.9 ± 23.5	82.7 ± 22.3	69.5 ± 26.1	69.2 ± 25.3
**BMI**	29.8 ± 6.6	29.9 ± 6.4	24.7 ± 8.1 &	24.6 ± 7.9 $
**Eutrophic (%)**	30.8	30.8	46.1	46.1
**Overweight (%)**	23.1	23.1	46.1	38.5
**Obese (%)**	46.1	46.1	7.8	15.4
**Muscle (kg)**	29.2 ± 6.3	28.9 ± 6.2	27.7 ± 9.8	27.8 ± 9.7
**Fat (%)**	34.7 ± 10.4	35.3 ± 10.6	27.0 ± 11.1 &	26.2 ± 10.4 $
**Glucose and insulin homeostasis**				
**Glucose (mg/dl)**	95.0 ± 12.2	94.2 ± 26.9	88.9 ± 11.0	89.8 ± 7.9
**HbA1 (%)**	5.2 ± 0.4	5.3 ± 0.5 *	5.30 ± 0.23	5.2 ± 0.3
**Insulin (µU/mL)**	10.6 ± 5.3	9.5 ± 3.5	27.4 ± 21.5 &	9.3 ± 4.8 *
**HOMA_IR**	2.5 ± 1.3	2.2 ± 0.7	6.46 ± 5.44 &	2.0 ± 0.9 *
**HOMA_beta**	170.5 ± 159.9	145.5 ± 125.5	372.3 ± 172.2 &	352.3 ± 239.7 $
**QUICKI**	0.34 ± 0.02	0.34 ± 0.02	0.32 ± 0.04	0.35 ± 0.02 *
**Adipo_IR**	11.0 ± 7.5	10.0 ± 5.6	27.2 ± 24.7 &	11.4 ± 10.1
**Biochemical measurements**				
**Leptin (ng/mL)**	39.7 ± 28.8	26.1 ± 19.9 *	11.7 ± 10.3 &	16.7 ± 17.7 $
**Adiponectin (mg/mL)**	3.0 ± 0.8	2.5 ± 1.3 *	4.6 ± 2.3 &	2.6 ± 1.2 *
**AST (U/L)**	25.8 ± 8.4	24.9 ± 7.3	32.4 ± 7.5 &	30.4 ± 5.0 $
**ALT (U/L)**	28.1 ± 16.4	26.2 ± 20.4	42.2 ± 23.9	31.7 ± 11.8 *
**GGT (U/L)**	23.1 ± 11.1	28.9 ± 24.9	27.0 ± 9.8	24.9 ± 10.7
**AST/ALT**	1.04 ± 0.4	1.19 ± 0.5	0.93 ± 0.4	1.07 ± 0.4
**Adipo/Lept**	0.23 ± 0.4	0.23 ± 0.4	1.26 ± 3.2 &	0.66 ± 1.2 $
**Lipid panel**				
**T_CHO (mg/dL)**	199.3 ± 38.4	190.7 ± 35.2	177.6 ± 43.7	161.1 ± 34.5 *$
**T_TG (mg/dL)**	151.5 ± 77.7	114.3 ± 61.6 *	119.1 ± 61.4	136.4 ± 84.1
**VLDL (mg/dL)**	30.3 ± 15.5	22.9 ± 12.3 *	23.8 ± 12.3	27.3 ± 16.8
**LDL-C (mg/dL)**	117.8 ± 31.5	110.6 ± 28.1	113.6 ± 36.3	97.6 ± 29.7 *
**HDL-C (mg/dL)**	53.0 ± 11.1	54.1 ± 12.1	41.1 ± 9.9 &	39.9 ± 8.6 $

Symbols represent statistical difference (*p* < 0.05) for paired *t*-test comparing post-exercise versus baseline (*) within each group and unpaired *t*-test comparing PLWH versus control subjects at baseline (&) and post-exercise ($).

## Data Availability

The original contributions presented in this study are included in the article/Supplementary Material. Further inquiries can be directed to the corresponding author. A preprint can also be accessed through MedRxiv (DOI https://doi.org/10.1101/2025.06.02.25328634).

## Supplementary Materials

The following supporting information can be downloaded at: https://www.mdpi.com/article/10.3390/metabo16010016/s1, Supplementary File, Lipid Data. Molar concentrations of all plasma lipid species (nmol/µL of plasma). Please see the main text for the description of lipid subclass abbreviations. Supplementary Figure S1. Venn diagram of the comparisons PLWH versus control subjects at baseline and post exercise (as illustrated in Figure 2). Please see the main text for abbreviations. Supplementary Figure S2. Results from multivariate analyses by principal component analysis (PCA) and partial least square discriminant analysis (PLSDA) showing score (A,C) and loadings (B,D) plots. Please see the main text for the description of lipid subclasses abbreviations. Supplementary Figure S3. Venn diagram of the comparisons post exercise versus baseline in control subjects and PLWH (as shown in Figure 3). Please see the main text for abbreviations. Supplementary Figure S4. Concentrations of four lipid species displaying > 10-fold reduction after exercise training in both control subjects and PLWH (as shown in Figure 3). Lines represent increasing (red) or decreasing (blue) concentrations from baseline to post exercise from the same individual. Please note that concentrations of 2H-Cer (d18:1/24:1) are in pmol/µL of plasma, whereas for other lipid species in nmol/µL of plasma. For abbreviations see main text. All data produced in the present study are available upon reasonable request to the authors.

## Abbreviations

The following abbreviations are used in this manuscript:

AC	Acylcarnitines
ADIPO_IR	Adipose Insulin Resistance Index
AIDS	Acquired immunodeficiency syndrome
ALT	Alanine Transaminase
ART	Antiretroviral therapy
AST	Aspartate Transaminase
BMI	Body Mass Index
CE	Cholesteryl Esters
Cer	Ceramides
CV	Coefficient of Variation
CVD	Cardiovascular diseases
DG	Diacylglycerides
FAT %	Body Fat Percentage
FC	Free cholesterol
FFA	Free Fatty Acid
GGT	Gamma glutamyl transferase
HbA1c	Hemoglobin A1c
H-Cer	Hexosyl Ceramide
HDL-C	High-Density Lipoprotein Cholesterol
HIV	Human Immunodeficiency Virus
HOMA_beta	Homeostasis Model Assessment of β-cell function
HOMA_IR	Homeostasis Model Assessment of Insulin Resistance
IDA	Information Dependent Acquisition
LDL-C	Low-Density Lipoprotein Cholesterol
LPC	Lyso-Phosphatidylcholine
LPE	Lyso-Phosphatidylethanolamine
MTBE	Methyl tert-butyl ether
oPC	Plasmanyl-Phosphatidilcholine
oPE	plasmanyl-Phosphatidylethanolamine
PC	Phosphatidylcholine
PCA	Principal Component Analysis
PCSK9	Proprotein Convertase Subtilisin/Kexin Type 9
PE	Phosphatidylethanolamine
PhE	Phytosterol esters
PI	Phosphatidylinositol
PLSDA	Partial Least Squares Discriminant Analysis
PLWH	People Living with HIV
pPC	Plasmenyl-Phosphatidilcholine
pPE	Plasmenyl-Phosphatidylethanolamine
Q10	Coenzyme Q-10
QUICKI	Quantitative Insulin Sensitivity Check Index
SM	Sphingomyelin
STD	Sexually Transmitted Disease
T_CHO	Total Cholesterol
T_TG	Total Triglycerides
TG	Triglycerides
TLE	Total Lipid Extract
TripleTOF	Triple quadrupole time-of-flight mass spectrometry
UHPLC	Ultra-High-Pressure Liquid Chromatography
VLDL	Very Low-Density Lipoprotein Cholesterol
