# Supplementary material for: The Effect of Short-Term and High-Intensity Functional Circuit Training on Plasma Lipidome Profiles of People Living with and Without HIV
Source: Metabolites. 2025 Dec 24;16(1):16. doi: 10.3390/metabo16010016 (PMC12844262; doi:10.3390/metabo16010016)
Supplement: Supplementary file 1 [file metabolites-16-00016-s001.zip › 25-06-2025 Supplemental_file1_Internal_Standards.pdf]

Table S1. Lipid subclasses and respective internal standards with their concentrations in 50 µL.

Lipid subclass	Internal Standard	in 50 µL (pM)
AC	AC (10:0)	2911.2
FFA	FFA (13:0)	4665.2
DG	DG (14:0/14:0)	4875.1
TG	TG (14:0/14:0/14:0)	3456.9
LPC	LPC (17:1)	984.9
PC, oPC, pPC	PC (17:0/17:0)	1640.2
PE, oPE, pPE	PE (17:0/17:0)	1736.0
PI	PG (17:0/17:0)*	1664.3
1H-Cer	1H-Cer (d18:1/17:0)	350.1
2H-Cer	2H-Cer (d18:1/17:0)	285.3
Sulf-Cer	Sulf-Cer (d18:1/17:0)	61.6
Cer	Cer (d18:1/17:0)	905.9
SM	SM (d18:1/17:0)	348.6
FC	deuterated FC (d7)	12732.7
CE	CE (17:0)	7823.4
Q-10, VitE**	Q-6	169.2

* a phosphatidylglycerol was used as internal standard and a factor of 0.65 relative to PI was calculated in dilution calibrations. ** the concentrations of VitE were normalized relative to coenzyme Q-6, and therefore should be considered non-quantitative.

Vocabulary

Acylcarnitines (AC), Free Fatty Acids (FFA), Diacylglycerols (DG), Triacylglycerols (TG), Lysophosphatidylcholines (LPC), Phosphatidylcholines (PC), Alkyl-phosphatidylcholines (oPC), Plasmenyl-phosphatidylcholines (pPC), Phosphatidylethanolamines (PE), Alkyl-phosphatidylethanolamines (oPE), Plasmenyl-phosphatidylethanolamines (pPE), Phosphatidylinositols (PI), Monohexosylceramides (1H-Cer), Dihexosylceramides (2H-Cer), Sulfatide ceramides (Sulf-Cer), Ceramides (Cer), Sphingomyelins (SM), Free Cholesterol (FC), Cholesteryl Esters (CE), Coenzyme Q10 (Q-10), Vitamin E (VitE)
