# Supplementary material for: The Effect of Short-Term and High-Intensity Functional Circuit Training on Plasma Lipidome Profiles of People Living with and Without HIV
Source: Metabolites. 2025 Dec 24;16(1):16. doi: 10.3390/metabo16010016 (PMC12844262; doi:10.3390/metabo16010016)
Supplement: Supplementary file 1 [file metabolites-16-00016-s001.zip › Figures S1-S4.pdf]

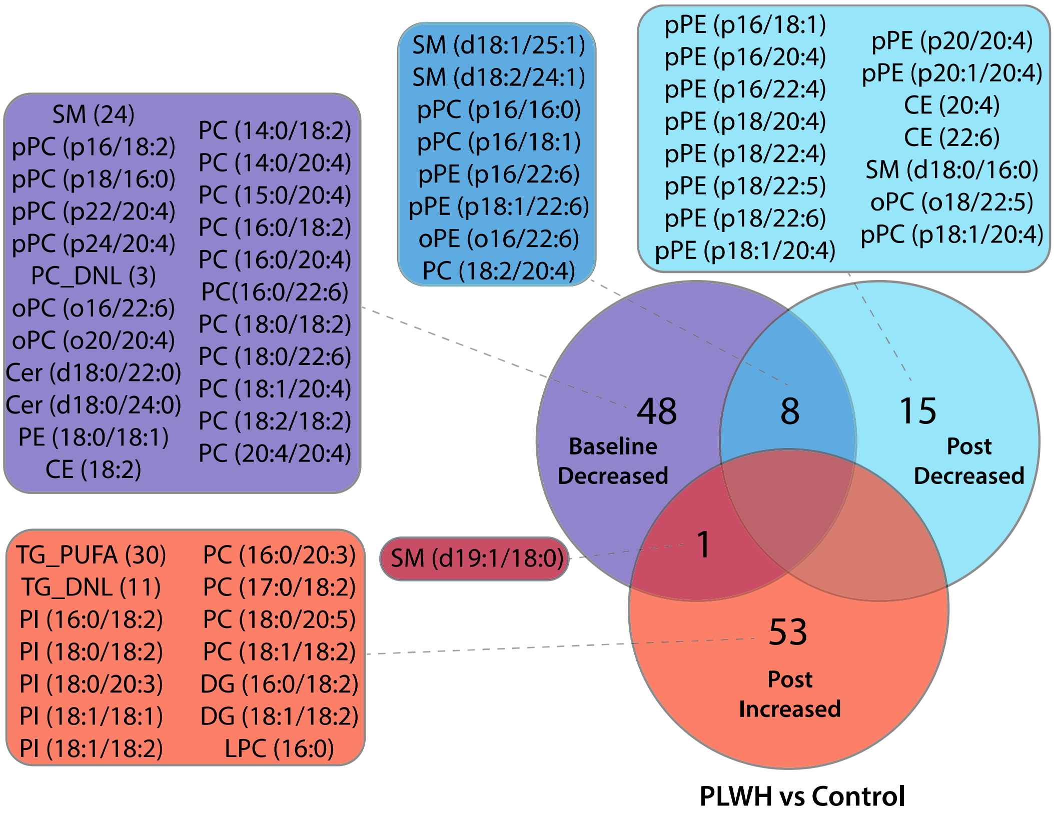

Figure S1. Venn diagram of the comparisons PLWH versus control subjects at baseline and post exercise (as illustrated in Figure 2). Please see the main text for abbreviations.

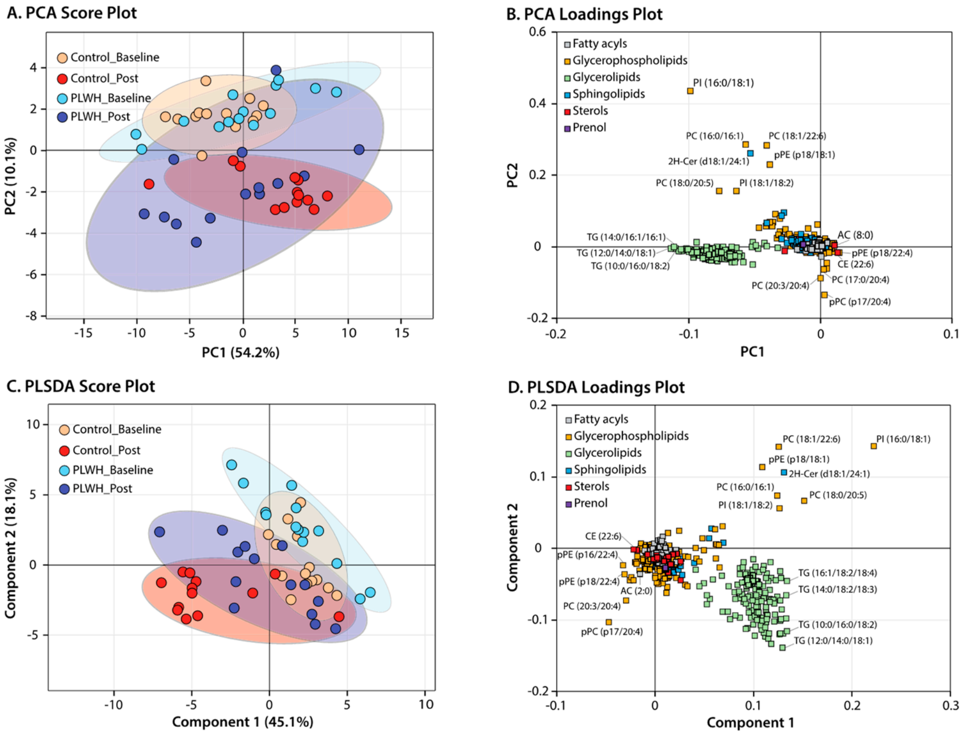

Figure S2. Results from multivariate analyses by principal component analysis (PCA) and partial least square discriminant analysis (PLSDA) showing score (A,C) and loadings (B,D) plots. Please see the main text for the description of lipid subclasses abbreviations.

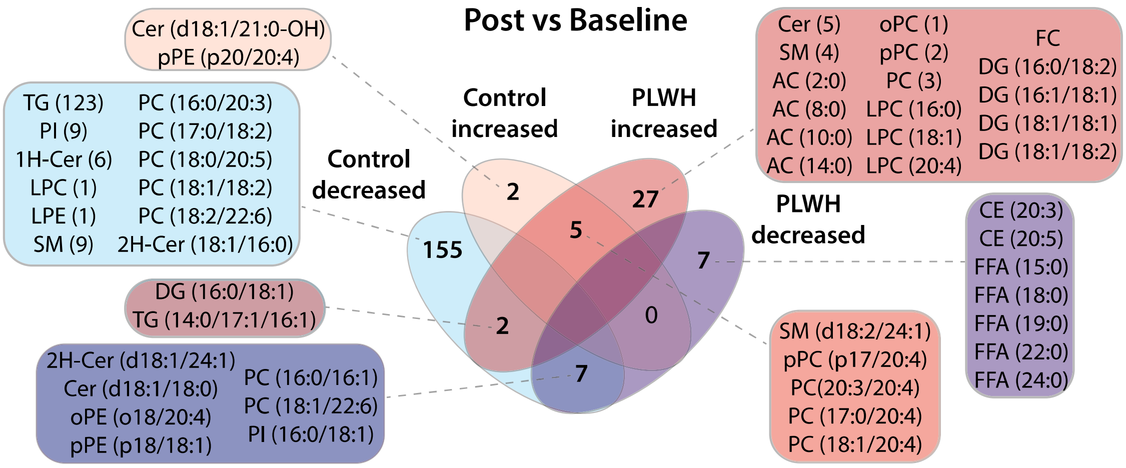

Figure S3. Venn diagram of the comparisons post exercise versus baseline in control subjects and PLWH (as shown in Figure 3). Please see the main text for abbreviations.

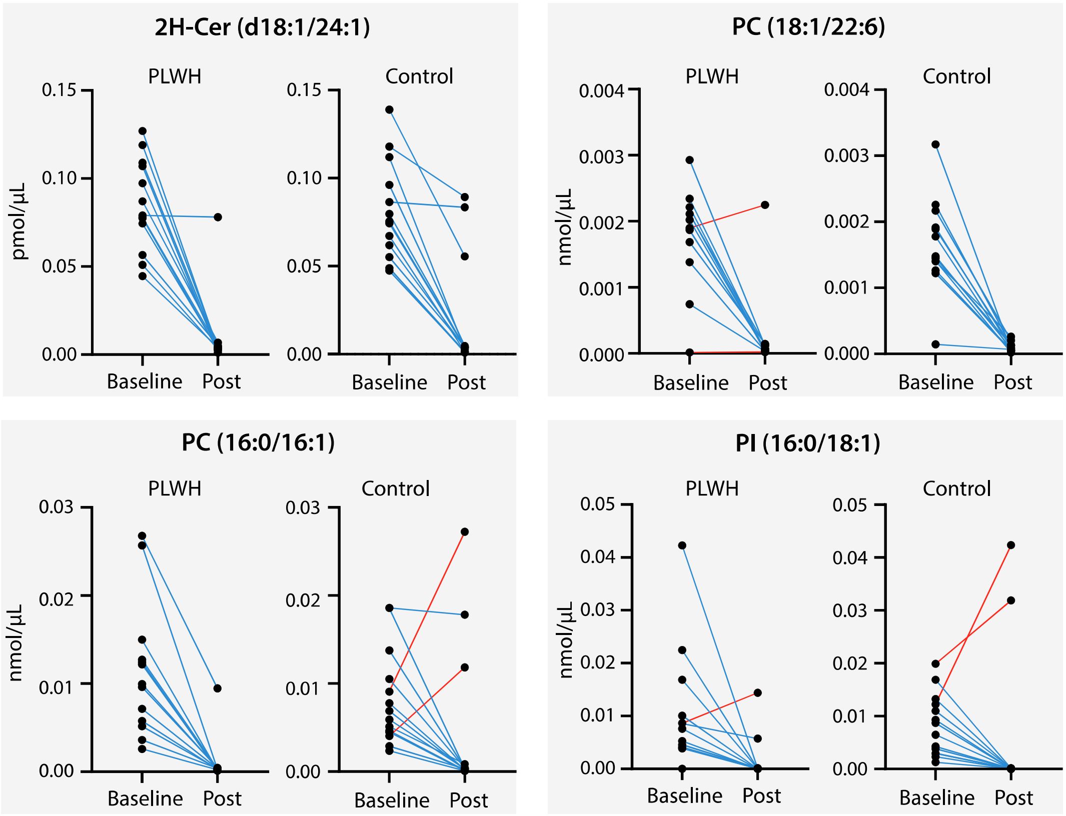

Figure S4. Concentrations of four lipid species displaying >10-fold reduction after exercise training in both control subjects and PLWH (as shown in Figure 3). Lines represent increasing (red) or decreasing (blue) concentrations from baseline to post exercise from the same individual. Please note that concentrations of 2H-Cer (d18:1/24:1) are in pmol/µL of plasma, whereas for other lipid species in nmol/µL of plasma. For abbreviations see main text.
